# A Pilot Prospective Study Evaluating the Effect of Curcuma-Based Herbal Food Supplement on the Outcome of In Vitro Fertilization in Patients Testing Positive for Four Immunological Biomarkers

**DOI:** 10.3390/medicina60020204

**Published:** 2024-01-25

**Authors:** Renato Colognato, Incoronata Laurenza, Gabriele Ersettigh, Giada Antonia Aiello, Marta Carnovali, Massimo Mariotti, Nicoletta Maxia

**Affiliations:** 1 Sanugen S.r.l., 21040 Gerenzano, VA, Italy; incolaurenza@yahoo.it; 2 Policlinico San Marco, 24046 Zingonia, BG, Italynicoletta.maxia@grupposandonato.it (N.M.); 3 Toma Institute S.r.l., 80139 Naples, NA, Italy; 4 IRCCS Ospedale Galeazzi-Sant’Ambrogio, 20157 Milano, MI, Italy; marta.carnovali@grupposandonato.it (M.C.); massimo.mariotti@unimi.it (M.M.); 5 Department of Biomedical, Surgical and Dental Sciences, University of Milan, 20122 Milano, MI, Italy

**Keywords:** in vitro fertilization, food supplement, biomarkers, pregnancy rate

## Abstract

*Background and Objectives:* Inflammation and oxidative stress have been described to reduce the chance for pregnancy instauration and maintenance. NOFLAMOX, a recently developed herbal preparation with recognized antioxidant and anti-inflammatory properties, can represent an interesting treatment to increase the chance of pregnancy, both physiological or after in vitro fertilization (IVF). The aim of this study was to assess NOFLAMOX’s effect; a population with unexplained infertility was screened for the recently described IMMUNOX panel based on four immunological biomarkers with a prospective study approach. *Materials and Methods*: Patients with unexplained infertility and positive for at least one of the biomarkers of the IMMUNOX panel were included in this study and treated with NOFLAMOX for three months prior to an IVF cycle. *Results*: Eighty-six patients (*n* = 86) were screened with the IMMUNOX panel and the forty-seven (54.5%) found positive were included in this study. In more detail, 11 were positive for TNFα (23.4%), 18 (38.3%) for glycodelin (GLY), 29 (61.7%) for Total Oxidative Status (TOS), and 32 (68.1%) for Complement Activity Toxic Factor (CATF). After three months of treatment, a significant reduction in the number of IMMUNOX-positive patients was observable, with 26 patients who turned IMMUNOX-negative displaying a quantitative statistically significant variation of 100% (11/11), 38.9% (7/18), 65.5% (18/29), and 75% (24/32), for TNFα, glycodelin, TOS, and CATF, respectively. Followed in the subsequent IVF cycle, this NOFLAMOX-treated population showed a pregnancy rate of 42.3% compared to the 4.7% of the IMMUNOX-positive group of patients. *Conclusions*: Taken together, the results of this study suggest that NOFLAMOX could represent an interesting option for those patients with unexplained infertility of inflammatory/oxidative origin. Further studies are needed to confirm these results and explore possible strategies to restore fertility in women with immune-mediated sterility.

## 1. Introduction

According to the WHO, infertility is a social concern in the female population worldwide, with several non-embryologic factors suggested to be responsible for failed in vitro fertilization (IVF). Most of the clinical conditions which have been suggested to be involved in infertility share the common ability to generate low medium-grade systemic inflammation and increase oxidative stress [1]. Such a combination is well known to be able to impair both embryo implantation and early growth [2]. 

Inflammation has been described to interfere with ovulation, corpus luteus formation, the endometrial cycle, mechanical aspects of implantation, maternal immunomodulation, endocrine asset, tubal lumen size, and other mechanisms relevant for pregnancy initiation [2]. 

Moreover, IVF techniques has been shown to trigger oxidative stress by influencing different parameters: First, the in vitro mechanical manipulation of eggs, sperm, and embryos itself induces free radical accumulation [3]. In addition, other factors such as the imbalance of cytokines/growth factors and change in chemical and physical parameters (pH, osmotic equilibrium, temperature, UV light) contribute to the generation of oxidative stress which, in conclusion, can be considered one of the most important factors that negatively affects IVF outcome [4]. Several data support the hypothesis that antioxidant treatments improve pregnancy rates after IVF techniques [5]. A recent report also suggests that treatment with antioxidant food supplements, starting three months before IVF cycles, reduces oxidative stress in the follicular fluid increasing the number of high-quality oocytes recovered at the pick-up [6]. 

We have recently described and published [7] panel of four biomarkers (IMMUNOX panel) composed of glycodelin (GLY), Tumor Necrosis Factor-α (TNFα), Complement Activation Toxic Factor (CATF), and Total Oxidative Status (TOS). This panel has been proposed as a new diagnostic criterion for unexplained infertility based on potential imbalance immunological parameters [7]. The biomarkers that have been proposed have been chosen for their direct correlation with different aspects of immune system activation and action. TNFα, a a “first phase” cytokine, has been introduced in clinical practices to monitor inflammatory conditions. The Total Oxidative Status reflects the oxidative stress due to the formation of reactive oxygen or nitrogen species because of over-activation of the immune response triggering potential host tissue damage (the embryo in our case) [7]. Moreover, the complement activation can be differently stimulated in patients, also because genetic susceptibility represents an important part of the immune response pathway, crucial in the early phase of embryo implantation [7]. Glycodelin, also known as placental protein 14 (pp14), is an endometrial glycoprotein induced by progesterone and correlated to the modulation of the maternal immune response to embryo antigens [7]. For these reasons, the IMMUNOX panel may represent an interesting cumulative panel, able to predict the risk of a significant number of immune-related infertility and pregnancy failure cases.

Herbal remedies with anti-inflammatory and antioxidant effects were introduced in clinical studies and practice many years ago with interesting results. Curcumin is a polyphenol extracted from the plant *Curcuma longa* which has been described to exert antioxidant, anti-inflammatory, anticancer, and neuroprotective activities. The efficacy of curcumin as an anti-inflammatory agent has been proved on human patients in clinical trials against rheumatoid arthritis, ulcerative colitis, and radiotherapy-induced dermatitis [8].

Bromelain is a protease extracted from the fruit or stem of the pineapple plant. It is known to reduce inflammation through inhibiting cyclo-oxygenase-2 expression and inactivating NFκB [9]. The presence of several other well-recognized pharmacological properties such as antithrombotic, fibrinolytic, anticancer, and immunomodulatory effects makes this compound suitable for clinical uses [10]. 

Piperine, the main constituent of *Piper nigrum* extract, stimulates intestinal absorption [11] and inhibits hepatic glucuronidation, a fundamental reaction for xenobiotic inactivation and excretion, thus ensuring better bioavailability of the other supplement molecules [12]. Curcumin, in fact, has poor bioavailability due to difficult intestinal absorption.

Papain, a serine protease extract from *Carica papaya*, has already been described as an anti-inflammatory enzyme in inflammatory bowel diseases [13] and wound healing [14]. 

A combination of these two plants with other herbal extracts may represent a suitable option to reduce inflammation and oxidation, and thus increase the chance of pregnancy after IVF. We have therefore studied the effects of a combination of these herbal remedies in regulating immune system activity in a population of women with unexplained infertility. 

## 2. Materials and Methods

### 2.1. Subjects

This study included patients who were referred to the EEIA/Clinica San Carlo (Paderno Dugnano (MI), Milan, Italy) with a diagnosis of unexplained infertility and who underwent a Controlled Ovarian Hyperstimulation cycle (COH). The Assisted Reproduction department runs over 400–500 IVF cycles per year.

The enrollment inclusion criteria were (i) eligibility for enrollment in a Controlled Ovarian Hyperstimulation cycle (COH) according to the clinic’s procedures, (ii) failure of at least two previous IVF cycles, (iii) diagnosis of unexplained infertility according to the 2015 guidelines of the Italian Ministry of Health (Art. 7-law n. 40/2004), (iv) body mass index (BMI) < 30 kg/m^2^, (v) age < 40 years, (vi) good ovarian reserve, defined on the basis of FSH and AMH values, (vii) physiologic range for estradiol and LH hormones, (viii) normal karyotype, and (ix) absence of uterine disease or congenital anomalies (i.e., fibroid, uterine polyp, uterine septum, uterus didelphus, etc.). Exclusion criteria were (i) ovarian and tubal dysfunction, (ii) BMI > 30, (iii) age > 40 years, (iv) poor stimulation response (≤5 eggs collected), and (v) poor embryo development (<2 high-quality embryos with LOW grade). 

The Ethical Committee of the EEIA/Clinica San Carlo (Paderno Dugnano (MI), Italy) approved this study. All the women provided signed informed consent before enrollment. 

### 2.2. Study Design

This was a single-center, observational, prospective clinical trial. Patients were screened with the IMMUNOX panel based on four immunological biomarkers [7], and those with at least a single biomarker outside physiologic range were enrolled for this study and subjected to a three-month treatment with NOFLAMOX, a patented food supplement, before undergoing an in vitro fertilization (IVF) cycle. After the treatment, a second IMMUNOX panel screening was carried out to evaluate NOFLAMOX’s efficacy. Those patients who had a normalization in the IMMUNOX panel were followed up during the next IVF cycle. NOFLAMOX treatment was continued until the result of the embryo transfer was assessed with β–HCG analysis. The primary endpoint of this study was to evaluate changes in altered immune biomarkers measured with the IMMUNOX panel after three months of treatment. 

The secondary endpoint was to evaluate the pregnancy rate in IMMUNOX-positive patients after the treatment. As previously described [7], a patient was considered IMMUNOX-positive when at least one of the evaluated markers (TNFα, GLY, TOS, and CATF) was out of the physiological range.

### 2.3. Biomarkers Analysis

An amount of 8.5 mL of blood was drawn from each patient using a Vacutainer collection tube for serological examination (BD-Vacutainer, SST2 Advance 367953). Blood was centrifuged to separate the serum and stored at −20 °C in 250 mL aliquots until the assays were performed. To avoid any potential loss of biomarker activity, the analyses were carried out on −20 °C aliquots stored on the same day or the day after the blood was drawn. Analysis of the three biomarkers (Glycodelin, TNFα, TOS) was carried out using commercial IVD-certified kits according to manufacturer protocol. A Glycodelin ELISA kit (BIOSERV) was used to analyze Glycodelin. A PerOx (TOS/TOC) kit (Immunodiagnostik) was used to quantify TOS as lipid peroxidation. TNFα evaluation was carried out with a TNFα ELISA kit (BD BioScience, UK). Regarding the CE-IVD biomarkers, the physiologic ranges that were considered are those described by the kit protocol: <8.10 pg/mL, <6 mg/dL, and <180 μmol/L for TNFα, Glycodelin, and TOS, respectively. CATF assay was performed as described [7].

### 2.4. Composition and Use of Herbal Supplement

The food supplement used in this study (brand name: NOFLAMOX^TM^) is an orally applied formulation composed of different plant extracts mixed with the following dosage: bromelain (1200 GDU) 645 mg, Curcuma (dry extract) 394.5 mg, papain (1:100) 375 mg, and black pepper (dry extract) 3.15 mg. The amounts in weight refer to three capsules, which is the daily dosage recommended. Administration was advised separate to meals and the treatment duration was 3 months before starting the IVF cycle.

### 2.5. IVF/ICSI Procedure and Sample Collection

All patients were treated with luteal-phase gonadotropin-releasing hormone agonist (GnRHa) protocol. Ovarian stimulation was induced with recombinant follicle-stimulating hormone and human menopausal gonadotropin. Human Chorionic Gonadotrophin (HCG) was administered when the dominant follicles (two or more) reached 18 mm in diameter. Oocytes were collected 36 h after HCG administration with a vaginal ultrasound-guided follicular aspiration. Mature oocytes were incubated at 37 °C in 5% CO_2_ and then inseminated with spermatozoa 4–6 h after oocyte retrieval for IVF according to the quality of sperm. The oocyte retrieval day was defined as day 0. Then, the embryos were scored on day 3 on a scale of 1–5 according to Scott’s scoring system. Embryos of grades 1 and 2 were defined as high-quality embryos. Two high-quality embryos were transferred into a competent endometrium (thickness > 8 mm) at day 3. No frozen eggs were used in this study. Clinical pregnancy was defined by the presence of a beating fetal heart on ultrasound examination.

### 2.6. Statistical Analysis

A descriptive analysis of the frequency of testing positive for the IMMUNOX panel before and after the three months treatment was carried out. To evaluate the efficacy of the treatment in reducing the number of IMMUNOX-positive patients, a comparison of the frequency before and after the treatment was carried out. The overall pregnancy rate after IVF was also reported for the subgroup of patients in which the IMMUNOX panel was negative after the treatment. Due to the pilot nature of this study, we did not carry out “a priori” analysis to estimate the sample size of this study. All statistical analyses were performed with MedCalc (v.12.1.4; MedCalc software BVBA). The χ^2^ test was performed to investigate comparisons of proportions. A *p* < 0.05 was considered statistically significant.

## 3. Results

### 3.1. Study Population

Eighty-six (n = 86) women referred to the EEIA/Clinica San Carlo (Paderno Dugnano, Milan, Italy) were screened with the IMMUNOX panel between June and October 2015 [7]. 

**Figure 1:**
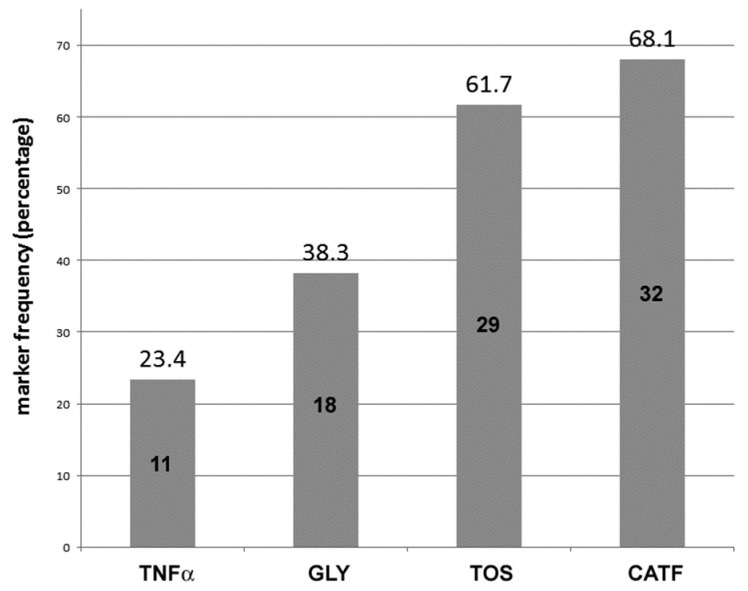
Percentage of unexplained infertility patients (*n* = 47) showing positivity for at least one of the biomarkers analyzed: TNFα (*n* = 11), GLY (*n* = 18), TOS (*n* = 29), and CATF (*n* = 32).

Forty-seven patients (n = 47) (54.5%) were found positive for the IMMUNOX panel and were enrolled in this study. Of these, eleven (*n* = 11) were positive for TNFα (23.4%), 18 (38.3%) for GLY, 29 (61.7%) for TOS, and 32 (68.1%) for CATF (Figure 1). 

No significant difference was observed for baseline characteristics (age, BMI, N. of previous IVF cycles) between IMMUNOX-positive and -negative patients (Table 1).

### 3.2. Modulation of Immunological Biomarkers after NOFLAMOX Treatment 

The impact of NOFLAMOX on oxidative and inflammatory status was assessed in the IMMUNOX-positive population after three months of treatment.

**Figure 2:**
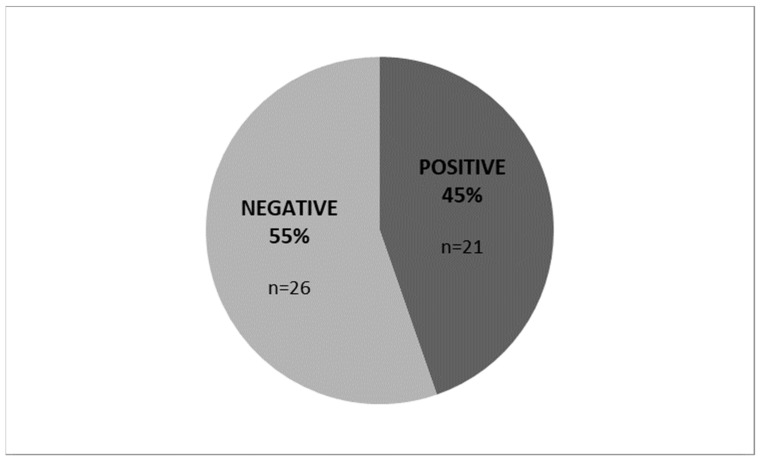
IMMUNOX analysis after NOFLAMOX treatment: 55.3% of IMMUNOX-positive population (*n* = 26) were IMMUNOX-negative after NOFLAMOX treatment.

Twenty-six (*n* = 26) patients out of forty-seven became IMMUNOX-negative after the treatment (55.3%; *p* < 0.05) (Figure 2) and were followed up during the next fertilization cycle. 

**Figure 3:**
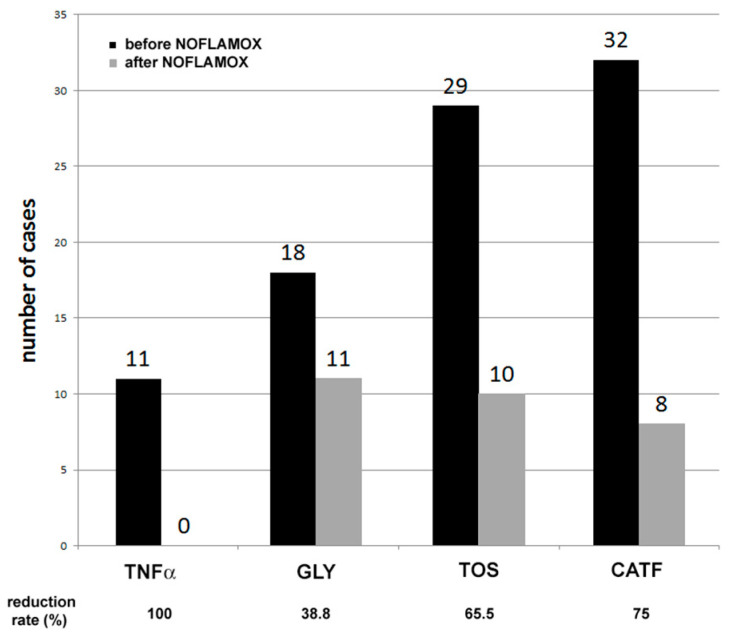
IMMUNOX single marker analysis after NOFLAMOX treatment. Number of positive cases before and after NOFLAMOX treatment of each biomarker and specific reduction rate.

To be more specific, the normalization rate was 100% (11/11), 38.9% (7/18), 65.5% (18/29), and 75% (24/32), for TNFα, glycodelin, TOS, and CATF, respectively (Figure 3). Next, the 26 patients who were IMMUNOX-negative were introduced into an IVF cycle program. NOFLAMOX treatment was continued until the result of the embryo transfer was assessed by β–HCG analysis.

### 3.3. Association between NOFLAMOX Treatment and Pregnancy Rate

The efficacy of NOFLAMOX was evaluated in terms of pregnancy rate in the subgroup of twenty-six (*n*=26) patients that had shown a normalization in the IMMUNOX biomarkers panel. After the treatment with NOFLAMOX, the pregnancy rate in this population was 42.3% (11 patients out of 26 enrolled women) compared to 4.76% in the IMMUNOX-positive group of patients (1 patient out of 21 enrolled women) (Table 2).

## 4. Discussion

The results of this study show the evidence of the efficacy of NOFLAMOX in reducing inflammatory/oxidative status triggering an increase of the pregnancy rate in women undergoing an IVF cycle after the normalization of the IMMUNOX-related biomarkers.

In this study, we retrospectively analyzed a cohort of patients with a diagnosis of unexplained infertility and who underwent a Controlled Ovarian Hyperstimulation cycle (COH). Before the treatment with NOFLAMOX, patients were screened with the IMMUNOX panel to select a subpopulation of women with evidence of inflammatory/oxidative changes. The IMMUNOX panel was recently proposed as a new diagnostic criterion for unexplained infertility and is composed of four biomarkers: Glycodelin (GLY), Tumor Necrosis Factor-α (TNFα), Complement Activation Toxic Factor (CATF), and Total Oxidative Status (TOS) [7]. Previous data suggest that the IMMUNOX panel may represent an interesting cumulative panel, able to predict both the risk of immune-related infertility and pregnancy outcome [7]. In a previous cohort of IMMUNOX-positive patients, the reported pregnancy rate after IVF was 2.9% [7], suggesting a strong correlation between these biomarkers and reproductive processes. 

Inflammation and oxidative damage are considered dangerous factors for the instauration and maintenance of pregnancy both in natural condition and after IVF treatments [15,16,17]. In this setting, antioxidants (endogenous and exogenous) can suppress this disease-associated oxidative stress, reducing the induced human tissues damage and thus increasing the chance of pregnancy [18]. 

Antioxidants from phytochemicals, botanicals, and herbal preparations can antagonize the redox-dependent activation of immune cells and the production of inflammatory mediators from T-lymphocytes and macrophages [19,20]. Such suppression of the redox and inflammatory events promotes the development of immune tolerance against host tissues like the embryo [7].

The results of this study show that a combination of herbal remedies with well-known antioxidant and anti-inflammatory proprieties was able, within a three-month treatment period, to reduce the number of IMMUNOX-positive patients. 

Such an observation is consistent with the well-known proprieties of NOFLAMOX’s active herbal ingredients: curcumin has been proved to down-regulate the secretion of TNFα in several trials, thus reflecting a downregulation of non-physiological immune cells [21]. Such an observation was further confirmed in this trial: of the 11 patients with a positive result for TNFα biomarkers at the first IMMUNOX test, none had a positive finding after the treatment. 

Bromelain has been recently used in association with N-acetyl cysteine and alpha-lipoic acid as an anti-inflammatory treatment in a murine model of endometriosis [22]. Piperidine increases the bioavailability of curcuma and bromelain in the blood, thus increasing their biological effects [23]. 

**Figure 4:**
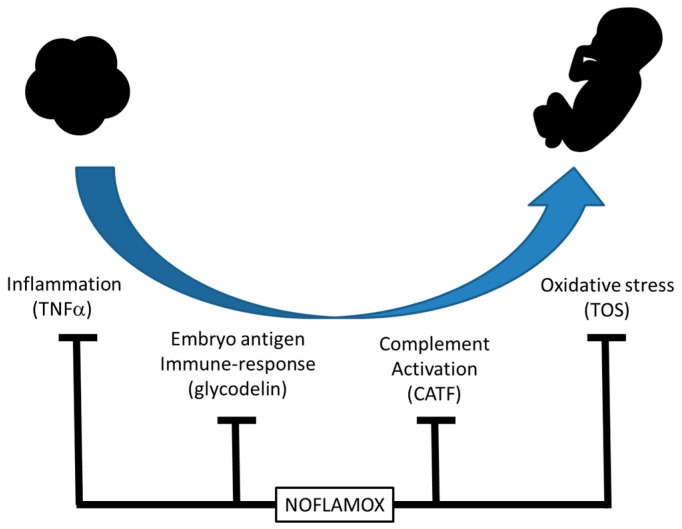
Schematic representation of the hypothetical immune-modulation effect of NOFLAMOX.

Such a combination of effects in the redox/inflammatory status has been proposed to have an effect during IVF FSH stimulation and within the period between the pick-up and the embryo transfer [7]. Such a hypothesis suggests that, by re-equilibrating the immunological conditions, NOFLAMOX could potentially improve embryo implantation and early fetal development by protecting the embryo from hyperactivity in the mother’s immune system [7]. The molecular mechanism is not understood in detail, but a wide immunomodulatory effect can be efficiently obtained with the correct administration of a food supplement containing active ingredients with anti-inflammatory and antioxidant capacity. In addition, the presence in the food supplement piperine facilitates the bioavailability of curcumin and papain; bromelain also generates a more rapid clearance of cytokines responsible for immune system activation maintenance. When the NOFLAMOX-dependent rebalancing of the immune system leads to pro-inflammatory cytokine (TNFα) downregulation, oxidative stress (TOS) reduction, complement activation suppression (CATF), and normalization of the glycodelin function, the embryo may be immunologically safe and free to develop (Figure 4). 

The results of this study provide an initial confirmation of this hypothesis: after three months of therapy, in the subgroup of patients who became IMMUNOX-negative, the overall pregnancy rate was 42.3%. Of interest is the fact that previous observations about IMMUNOX-positive patients had shown an overall pregnancy rate in IVF of 2.9% [7], whereas in this study, the success rate was 4.7%, suggesting a significant improvement in terms of IVF efficacy. 

Taken together, the results of this study suggest that part of idiopathic/unexplained infertility and IVF failure could be related to immunological disorders, thus confirming previously reported observations on the IMMUNOX panel [7]. It was also shown that the IMMUNOX panel represents an important tool to select a subgroup of patients with a systemic condition of inflammation/oxidative stress that may prevent embryo implantation and development. 

Finally, the results of this study provide the first piece of evidence that a combination of herbal supplements capable of reducing systemic inflammation/oxidative stress may increase the chance of pregnancy after IVF in this population. Other approaches like a personalized diet may be effective as well; nevertheless, it is not suitable for clinical studies, whereas nutraceutical administration can be better included in a clinical protocol.

This study has some limitations which should be highlighted: Because of the pivotal nature of this study, we did not estimate an a priori sample size. Similarly, we were unable to retrieve information about the pregnancy status of the patients who had an IMMUNOX-negative panel. It is of interest to note, however, that the differences observed in the overall pregnancy rate vs. the pregnancy rate in the previously described cohort with a positive IMMUNOX panel [7] strongly suggests the robustness of present findings. Additional, larger, and controlled studies on NOFLAMOX will help to clarify this point.

## 5. Conclusions 

Taken together, the results of this study strongly suggest that NOFLAMOX could represent an interesting option for those patients with unexplained infertility of inflammatory/oxidative origin. 

Further studies are needed to confirm these results and to explore possible strategies to restore fertility in women with immune-mediated sterility.

## 6. Patents

NOFLAMOX food supplement is patented with number P020746. IMMUNOX test is patented with number P020742.

## Figures and Tables

**Table 1:** Population characteristics (*n* = 86).

Characteristic	Data
Age	36 ± 4
Body Mass Index	25 ± 3.5
Years of Infertility	4.4 ± 1.8
Number of IVF cycles	4.5 ± 1.8

**Table 2:** Pregnancy rate after NOFLAMOX treatment. A 42.3% chance of success can be measured in the IMMUNOX-negative population whereas the pregnancy rate in IMMUNOX-positive patients was 4.7%.

		N° Embryo Transfer		
		Positive	Negative	Pregnancy Rate (%)
**IMMUNOX**	Positive (n = 21)	1	20	4.76
	Negative (n = 26)	11	15	42.3
